# WRKY genes provide novel insights into their role against *Ralstonia solanacearum* infection in cultivated peanut (*Arachis hypogaea* L.)

**DOI:** 10.3389/fpls.2022.986673

**Published:** 2022-09-20

**Authors:** Lei Yan, Haotian Jin, Ali Raza, Yang Huang, Deping Gu, Xiaoyun Zou

**Affiliations:** ^1^Institute of Crops, Jiangxi Academy of Agricultural Sciences, Nanchang, China; ^2^College of Agriculture, Oil Crops Research Institute, Fujian Agriculture and Forestry University, Fuzhou, China

**Keywords:** biotic stress, gene structure, oilseed crop, phylogenetic analysis, expression analysis, bacterial wilt

## Abstract

As one of the most important and largest transcription factors, WRKY plays a critical role in plant disease resistance. However, little is known regarding the functions of the WRKY family in cultivated peanuts (*Arachis hypogaea* L.). In this study, a total of 174 WRKY genes (*AhWRKY*) were identified from the genome of cultivated peanuts. Phylogenetic analysis revealed that AhWRKY proteins could be divided into four groups, including 35 (20.12%) in group I, 107 (61.49%) in group II, 31 (17.82%) in group III, and 1 (0.57%) in group IV. This division is further supported by the conserved motif compositions and intron/exon structures. All *AhWRKY* genes were unevenly located on all 20 chromosomes, among which 132 pairs of fragment duplication and seven pairs of tandem duplications existed. Eighteen miRNAs were found to be targeting 50 *AhWRKY* genes. Most *AhWRKY* genes from some groups showed tissue-specific expression. *AhWRKY46, AhWRKY94, AhWRKY156, AhWRKY68, AhWRKY41, AhWRKY128, AhWRKY104, AhWRKY19, AhWRKY62, AhWRKY155, AhWRKY170, AhWRKY78, AhWRKY34, AhWRKY12, AhWRKY95*, and *AhWRKY76* were upregulated in ganhua18 and kainong313 genotypes after *Ralstonia solanacearum* infection. Ten *AhWRKY* genes (*AhWRKY34, AhWRKY76, AhWRKY78, AhWRKY120, AhWRKY153, AhWRKY155, AhWRKY159, AhWRKY160, AhWRKY161*, and *AhWRKY162*) from group III displayed different expression patterns in *R. solanacearum* sensitive and resistant peanut genotypes infected with the *R. solanacearum*. Two AhWRKY genes (*AhWRKY76* and *AhWRKY77*) from group III obtained the LRR domain. *AhWRKY77* downregulated in both genotypes; *AhWRKY76* showed lower-higher expression in ganhua18 and higher expression in kainong313. Both *AhWRKY76* and *AhWRKY77* are targeted by ahy-miR3512, which may have an important function in peanut disease resistance. This study identified candidate *WRKY* genes with possible roles in peanut resistance against *R. solanacearum* infection. These findings not only contribute to our understanding of the novel role of *WRKY* family genes but also provide valuable information for disease resistance in *A. hypogaea*.

## Introduction

Transcription factors (TFs) activate diverse signal transduction cascades and regulate the transcriptional mode of targeted genes, which helps crop plants to adapt to diverse environmental stresses (Javed et al., 2020, 2022; Zhu et al., 2021; Haider et al., 2022). Among various TFs, WRKY TFs are one of the largest TF families in plants and regulate various biological processes, including growth, development, stress, and disease resistance (Rushton et al., 2010; Zhu et al., 2021; Javed et al., 2022). WRKY proteins contain a highly conserved WRKYGQK motif at N-terminus and a zinc finger motif (C_2_H_2_: CX_4_CX_22−23_HX_1_H, C_2_HC: CX_7_CX_23_HX_1_C) at C-terminus (Rushton et al., 2010). WRKY proteins can be classified into four groups (I, II, III, and IV). Group I members have two WRKY domains and the C_2_H_2_ type zinc finger. Group II and Group III members have one WRKY domain; Group II members have a C_2_H_2_ type zinc finger and Group III members have a C_2_HC-type. Group I can be classified into two subgroups Ia and Ib based on two WRKY domains at the N and C terminus (Chen et al., 2019a; Li Z et al., 2020). Group II WRKY can be further classified into five subgroups (IIa, IIb, IIc, IId, and IIe) based on the sequence of the DNA-binding domain (Zhang and Wang, 2005). Group III is separated into subgroups IIIa and IIIb based on the zinc-finger motif structure (Eulgem et al., 2000; Chen et al., 2019a). Group IV has an incomplete or partial WRKY domain and lacks a zinc-finger motif, suggesting that members of this group may have lost their function as WRKY TFs (Li Z et al., 2020; Javed et al., 2022).

*Ralstonia solanacearum* is a soil-borne bacterium; it usually infects plants through roots and then spreads through the vascular system to the whole plant, eventually causing mechanical blockage of the water transport system. In response to bacterial wilt (BW) disease, plants become wilt and die when the leaves are still green (Hikichi et al., 2017). A previous study indicated that *AtWRKY52* (group III member) in *Arabidopsis thaliana* confers resistance to the bacterial soil-borne pathogen *R. solanacearum* (Deslandes et al., 1998, 2002). *CaWRKY28, CaWRKY30* (group III member), *CaWRKY40, CaWRKY41* (group III member), and *CaWRKY58* in pepper regulated the defense response to *R. solanacearum* in plants (Wang et al., 2012; Muhammad et al., 2018; Dang et al., 2019; Hussain et al., 2021; Liu et al., 2021; Yang et al., 2021). Overexpression of sugarcane class III *WRKY5* increased bacterial wilt resistance (Wang et al., 2020). Group III *SiWRKY53* is a source of resistance to BW in *Solanum incanum* L. (Mishra et al., 2021). WRKY, especially group III members, play a vital part in the BW resistance of plants.

So far, WRKY TF families have been identified in many plants. For instance, there are 74 *WRKY* genes in *Arabidopsis* (*Arabidopsis thaliana* L.) (Dong et al., 2003), 153 *WRKY* genes in rapeseed (*Brassica napus* L.) (He et al., 2016), 287 *WRKY* genes in banana (*Musa acuminata* L.) (Kaliyappan et al., 2016), 88 *WRKY* genes in common bean (*Phaseolus vulgaris* L.) (Wu et al., 2017), 54 *WRKY* genes in pineapple (*Ananas comosus* L.) (Xie et al., 2018), 97 *WRKY* genes in Actinidia (*Caragana intermedia* L.) (Wan et al., 2018), 94 *WRKY* genes in sorghum (*Sorghum bicolor* L.) (Baillo et al., 2020), and 224 *WRKY* genes in Camelina (*Camelina sativa* L.) (Song et al., 2020). Previously, 77 and 75 WRKY proteins have been identified from the two wild ancestral diploid genomes of cultivated tetraploid peanuts, *Arachis duranensis* and *Arachis ipaënsis* (Song et al., 2016). Recently, a study identified 158 WRKY proteins from cultivated tetraploid peanuts using an older version of *A. hypogea* and gene expression of *AhWRKY* family members in response to drought stress (Zhao et al., 2020).

Climate change and crop production are directly correlated with each other, and current climate changes significantly impact agricultural productivity (Sharma et al., 2021, 2022; Farooq et al., 2022). Mainly, different biotic diseases and abiotic environmental factors greatly affect the productivity and overall quality of various crop plants around the world (Irfan et al., 2016, 2021; Kumar et al., 2016; Jiang et al., 2017; Ansari et al., 2022; Raza et al., 2022a,b). Peanut (*Arachis hypogaea* L.) is one of the most important oil crops in the world, but its yield is severely limited by various biotic and abiotic stresses (Mishra et al., 2015; Gangurde et al., 2019). Among these, BW is the major biotic factor that significantly hampers peanut production in the field environment (Jiang et al., 2017). The BW is one of the most important diseases in peanuts, especially destructive in China. When infected with the *R. solanacearum*, peanut plants wilt and die. It usually causes a 400,000 hm^2^ incidence area and 10–30% yield loss in 1 year in China (Jiang et al., 2017). This study identified 174 WRKY proteins, comprehensive analysis of gene structures, chromosome location, evolution relationship, *cis*-acting element, putative miRNAs, and expression analysis of gene response to *R. solanacearum*. These results provide insights into the evolution of the peanut WRKYs and their novel functions in peanut BW resistance. The identification and characterization of these *WRKY* genes may provide opportunities for peanut disease improvement and breeding programs in the era of climate change.

## Materials and methods

### Identification of the WRKY genes in peanuts

The protein data of cultivated peanuts (cv. Tifrunner) were obtained from the peanut genome database (PeanutBase, https://www.peanutbase.org/). The HMM file of the WRKY domain (PF03106) was retrieved from the Pfam database (http://pfam.sanger.ac.uk/) and was used to search the WRKY family proteins in the peanut protein database with an E-value threshold of 1 × 10^−5^ by simple HMM search TBtools (Toolbox for Biologists) v1.098 (Chen et al., 2020). Arabidopsis WRKY protein sequences were downloaded from the database of The Arabidopsis Information Resource (TAIR, https://www.arabidopsis.org/). The protein sequences of AhWRKY and AtWRKY were subjected to BLAST alignment by TBtools (Toolbox for Biologists) v1.098 (*E* value <1 × 10^−5^) (Chen et al., 2020). Subsequently, all non-redundant peanut WRKY protein sequences were validated for the presence of the WRKY domain by submitting them as search queries to the NCBI CDD (http://www.ncbi.nlm.nih.gov/cdd/) and SMART databases (http://smart.embl.de/). Finally, 174 AhWRKY sequences were identified and named AhWRKY1 to AhWRKY174 according to their physical locations on the chromosome.

### Phylogenetic analysis

Multiple sequence alignment of AhWRKY proteins was conducted by MUSCLE with default parameters. A neighbor-joining (NJ) tree was constructed in MEGA 7.0 with the following criteria: Poisson model, pairwise deletion, and 1,000 bootstrap replications. Maximum likelihood (ML) analysis of the *AtWRKY* and *AhWRKY* gene family was conducted to draw a phylogenetic tree.

### Gene structure, chromosomal distribution, and gene duplication

The MEME online program (Multiple Expectation Maximization for Motif Elicitation: http://meme-suite.org/tools/meme/) was used to identify conserved motifs in the AhWRKY proteins with a maximum motif number of 10. Information on the intron–exon structure was obtained from the peanut genome database (*A. hypogaea* cv. Tifrunner) and the TBtools software was used for visualization (Chen et al., 2020). TBtools was also used to determine the chromosomal location of the *AhWRKY* genes and draw the image. The duplication types and collinear blocks were discovered and were analyzed using One Step MCScanX and Simple Ka/Ks calculator (NJ) of Tbtools, and a diagram of gene duplication events was drawn using the Tbtools. Duplication and divergence time was calculated by the following formula as described by Bertioli et al. (2016):


(1) $$\begin{array}{r} T=Ks/2\lambda \left(\lambda =8.12\times \;10^{-9}\right). \end{array}$$

### Physiochemical parameters and promoter analysis of *AhWRKY*

The molecular weight (Mw) and isoelectric point (pI) of the full-length proteins were predicted using the pI/Mw tool (https://web.expasy.org/compute_pi/) in ExPASy (Gasteiger et al., 2003). The 2,000-base-pair (bp) sequences upstream of the start codon ATG of *AhWRKY* genes were retrieved from the peanut genome using TBtools (Chen et al., 2020) and were submitted to the online software PlantCARE to identify the *cis*-acting elements. The draft of the element distribution on each promoter was sketched using TBtools.

### Prediction of putative miRNA targeting *AhWRKY* genes

The CDS of *AhWRKY* genes were employed to distinguish target miRNAs in the psRNATarget database (https://www.zhaolab.org/psRNATarget/home) with default parameters. The interaction network figure among the miRNAs and target genes was developed with the Cytoscape software (V3.8.2; https://cytoscape.org/download.html).

### Gene expression analysis

The expression data of the *AhWRKY* genes in different tissues were identified using RNA-seq (Tifrunner variety) data obtained from the PeanutBase database (Clevenger et al., 2016). The read counts were transformed to TPM (Transcripts Per Kilobase of exon model per Million mapped reads) by GenomicFeatures in R, and the heatmap diagram was constructed with lg (TPM+1) using TBtools.

Two peanut cultivars (ganhua 18, resistant; and kainong 313, susceptible) were used for expression analysis against BW disease. Plant growth and *R. solanacearum* inoculation were carried out according to Chen et al. (2014). Plants were grown in 10 cm × 10 cm pots, and every pot contained three seedlings. After 21 days of sowing seeds, they were watered with 20 mL of *R. solanacearum* strain race 1 suspension (10^7^ cfu ml^−1^) in one pot. Roots of three individual seedlings were sampled at 0, 6, 12, 24, 48, 72, and 96 h post-inoculation. The samples were frozen in liquid nitrogen immediately and stored at −80°C for further analysis.

The TianGen kit (TianGen, Beijing, China) was used to extract total RNA from roots, and the experimental operations were carried out according to the developer's instructions. Approximately, 5 μg of total RNA was reverse-transcribed using the RevertAid First Strand cDNA Synthesis Kit (Thermo Fisher Scientific, USA). The primers were designed using the Primer 5.0 software (Supplementary Table 4), and the quantitative real-time qRT-PCR was performed on LightCycler 96 (Roche) using the Aidlab SYBR Green Mix kit (Aidlab, Beijing, China). The PCR protocol was conducted with a 10 μL volume, which contained 0.5 μL of upstream and downstream primers (10 μmol L^−1^), 2 μL of cDNA template, 2 μL of double-distilled water (ddH_2_O), and 5 μL of 2 × SYBR (SYBR Green qPCR Mix). The PCR program was 95°C for 120 s, followed by 40 cycles of 95°C for 5 s and 60°C for 30 s. qRT-PCR was performed with three technical repetitions, and the relative expression level was calculated using the 2^−Δ*ΔCT*^ method.

## Results

### Identification of the *AhWRKY* family

In this study, a total of 174 WRKY sequences were identified in cultivated peanuts (cv. Tifrunner) and named *AhWRKY1* to *AhWRKY174* according to their physical locations on the chromosome (Supplementary Table 1). The *AhWRKY* genes vary in length from 498 bp (*AhWRKY22* and *AhWRKY31*) to 10,044 bp (*AhWRKY59*), and their coding proteins range from 91 (*AhWRKY22*) to 1,345 (*AhWRKY59*) amino acids. The MWs of AhWRKYs were between 10.4 (AhWRKY22) and 148.6 kDa (AhWRKY132), with the majority of them ranging from 20 to 85 kDa in peanuts. The predicted pI ranged from 4.88 (AhWRKY55) to 10.08 (AhWRKY132), with an average of 7.06, among which 67 AhWRKYs had pI > 7, and 107 AhWRKYs had pI <7.

### Phylogenetic analysis of the *AhWRKY* family

**Figure 1:**
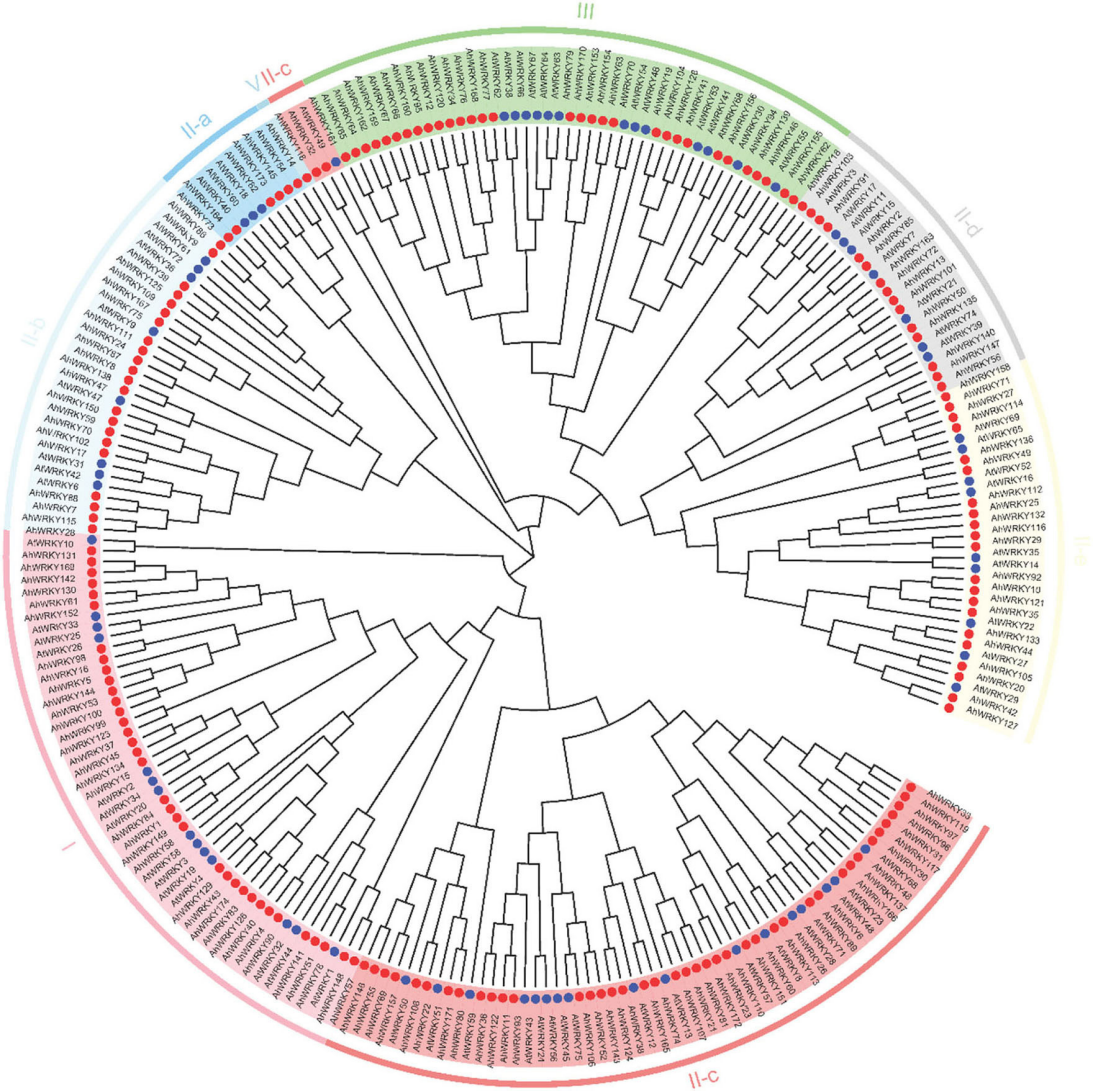
Phylogenetic tree of WRKY in *A. hypogaea* cv. Tifrunner and Arabidopsis. All full-length protein sequences were aligned by MUSCLE, and the tree was generated using the neighbor-joining (NJ) method in MEGA7 software. Different colors indicated groups I, II-a, II-b, II-c, II-d, II-e, III, and IV subfamily WRKY proteins from peanuts. The blue and red circles indicate the *Arabidopsis thaliana* and *A. hypogea* protein.

To explore the evolutionary relationship between AhWRKY TFs, a phylogenetic tree was constructed using 174 AhWRKY proteins in peanuts and 74 AtWRKYs from *Arabidopsis* (Figure 1). In brief, 174 WRKY TFs were divided into four groups, group I (35), group II (107), group III (31), and group IV (1); group II was further divided into five subgroups, subgroup II-a (6), subgroup II-b (22), subgroup II-c (43), subgroup II-d (15), and subgroup II-e (21) (Figure 1; Supplementary Table 1).

### Chromosomal mapping and duplication analysis of the *AhWRKY*

**Figure 2:**
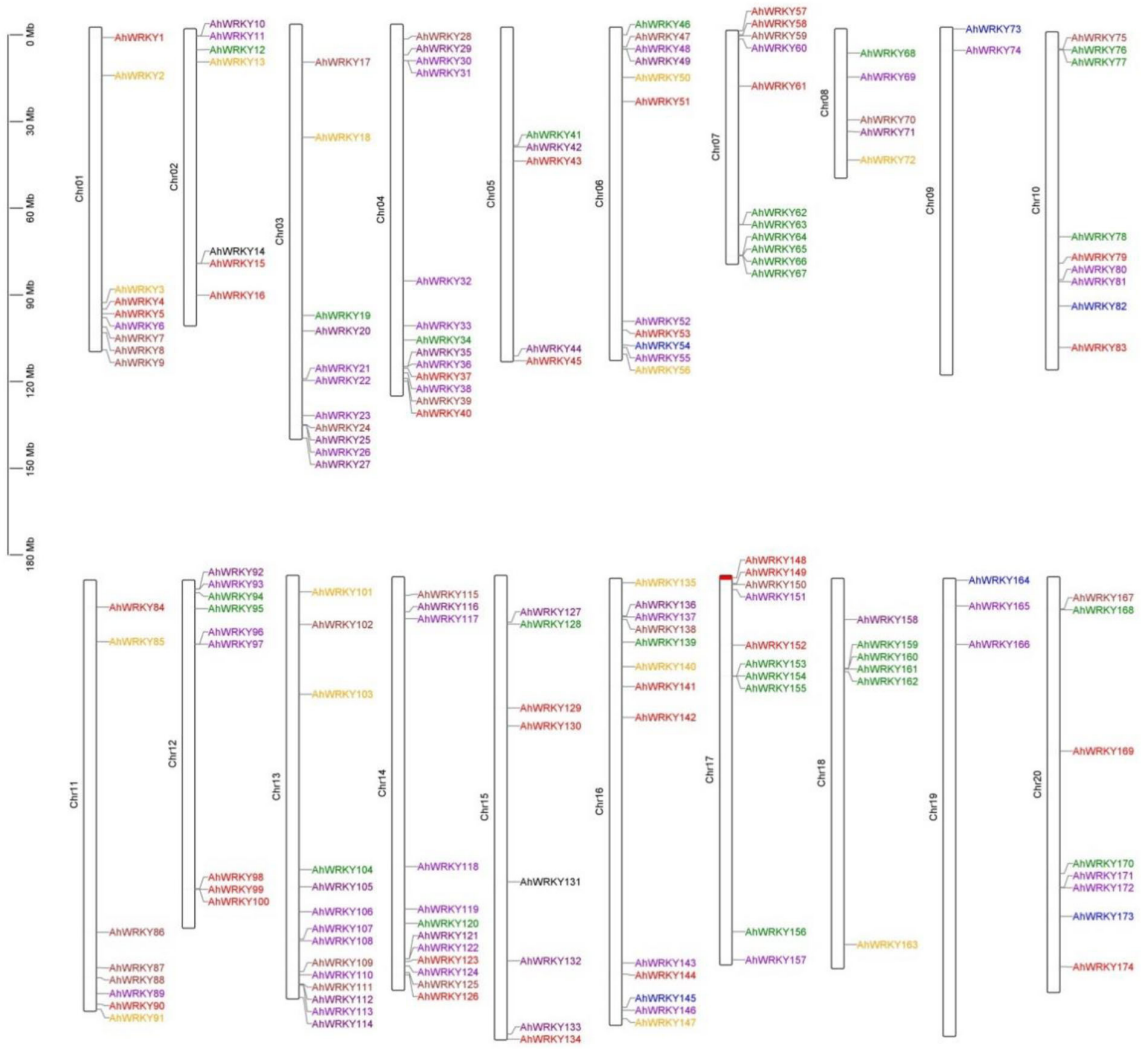
Chromosome mapping of *AhWRKY* genes. The scale represents a 30-Mb chromosomal distance. Red, sky blue, brown, DarkViolet, orange, purple, green, and blue indicated groups I, II-a, II-b, II-c, II-d, II-e, III, and IV, respectively.

The mapping results showed that 174 *AhWRKYs* were found to be unevenly distributed on 20 chromosomes using genome chromosomal location analysis (Figure 2). Most genes were present on chromosomes 4 and 13, whereas 16 genes belonging to the A and B genomes, respectively, accounted for 7.47% (13 genes) and 8.05% (14 genes), 7.47% (13 genes) of the total gene numbers, and the fewest genes were scattered on chromosome 9 from the A genome and chromosome 19 of the B genome, accounting for 1.15% (2 genes) and 1.72% (3 genes), respectively.

Chromosomal mapping showed there are many gene clusters of AhWRKY in different chromosomes. For example, *AhWRKY3-9* on chr1, *AhWRKY23-27* on chr3, *AhWRKY35-40* on chr4, *AhWRKY47-49* on chr6, *AhWRKY62-67* on chr7, *AhWRKY75-77* on chr10, *AhWRKY98-100* on chr12, *AhWRKY136-139* on chr16, *AhWRKY153-155* on chr17, and *AhWRKY159-162* on chr18.

**Figure 3:**
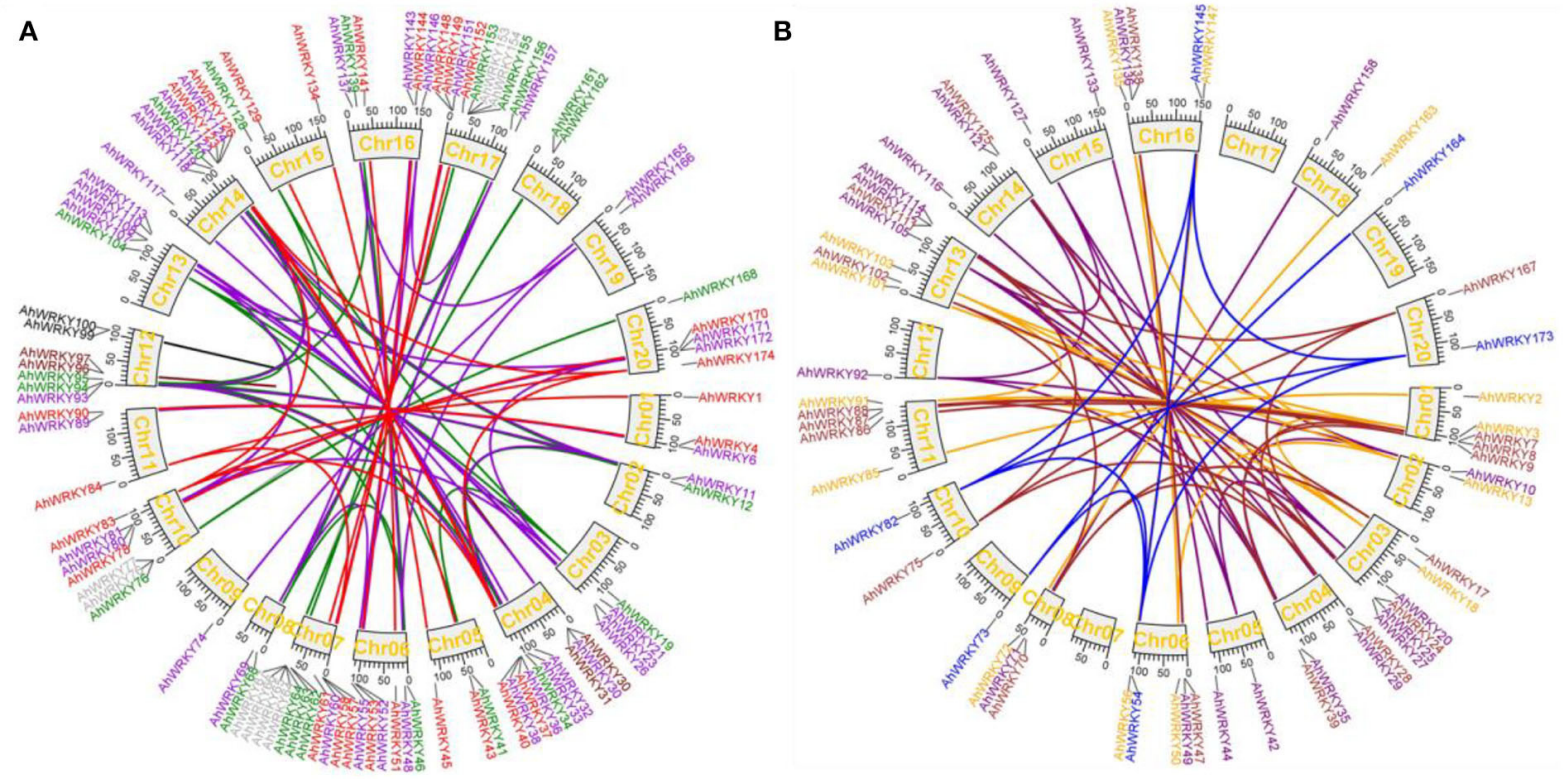
Circos figures for chromosome locations with AhWRKY duplication links. **(A)** Groups I, II-c, and III subfamily duplication links. Red, dark violet, and green lines indicated groups I, II-c, and III subfamily segmented, and black, maroon, and dark gray lines indicated groups I, II-c, and III subfamily tandem duplicated gene pairs, respectively. **(B)** Groups II-a, II-b, II-d, and II-e subfamily duplication links. Blue, brown, orange, and purple lines indicated group II-a, II-b, II-d, and II-e subfamily segmented duplicated gene pairs, respectively.

Collinearity analysis showed that among 174 *AhWRKYs*, 132 pairs of segmental duplication genes were identified; these were divided into seven groups according to the phylogenetic tree (Figure 3). Maximum segmental duplication events occurred in group II-c (34 pairs), followed by group III (22 pairs), group II-e (19 pairs), group II-b (18 pairs), group I (18 pairs), group II-d (13 pairs) and group II-a (8 pairs). Seven pairs of tandem duplication genes were detected, such as *AhWRKY99* and *AhWRKY100* in group I (Figure 3), *AhWRKY30* and *AhWRKY31, AhWRKY96* and *AhWRKY97* in group II-c, *AhWRKY64* and *AhWRKY65, AhWRKY66* and *AhWRKY67, AhWRKY76* and *AhWRKY77*, and *AhWRKY153* and *AhWRKY154* in group III. The Ka/Ks for segmental duplication was 0.05–1.21 with an average of 0.31, while the ratio of tandem duplication ranged from 0.42 to 0.94 with an average of 0.63. Only the Ka/Ks ratio of one pair (*AhWRKY119* and *AhWRKY33*) was 1.21, which indicated the gene pair was subjected to positive selection, and others were strongly subjected to pure selection. These segmental and tandem duplications may occur in 0.34–186.58 and 6.11–35.81 Mya, respectively (Figure 3; Supplementary Table 2).

### Structure analysis of *AhWRKY* genes

**Figure 4:**
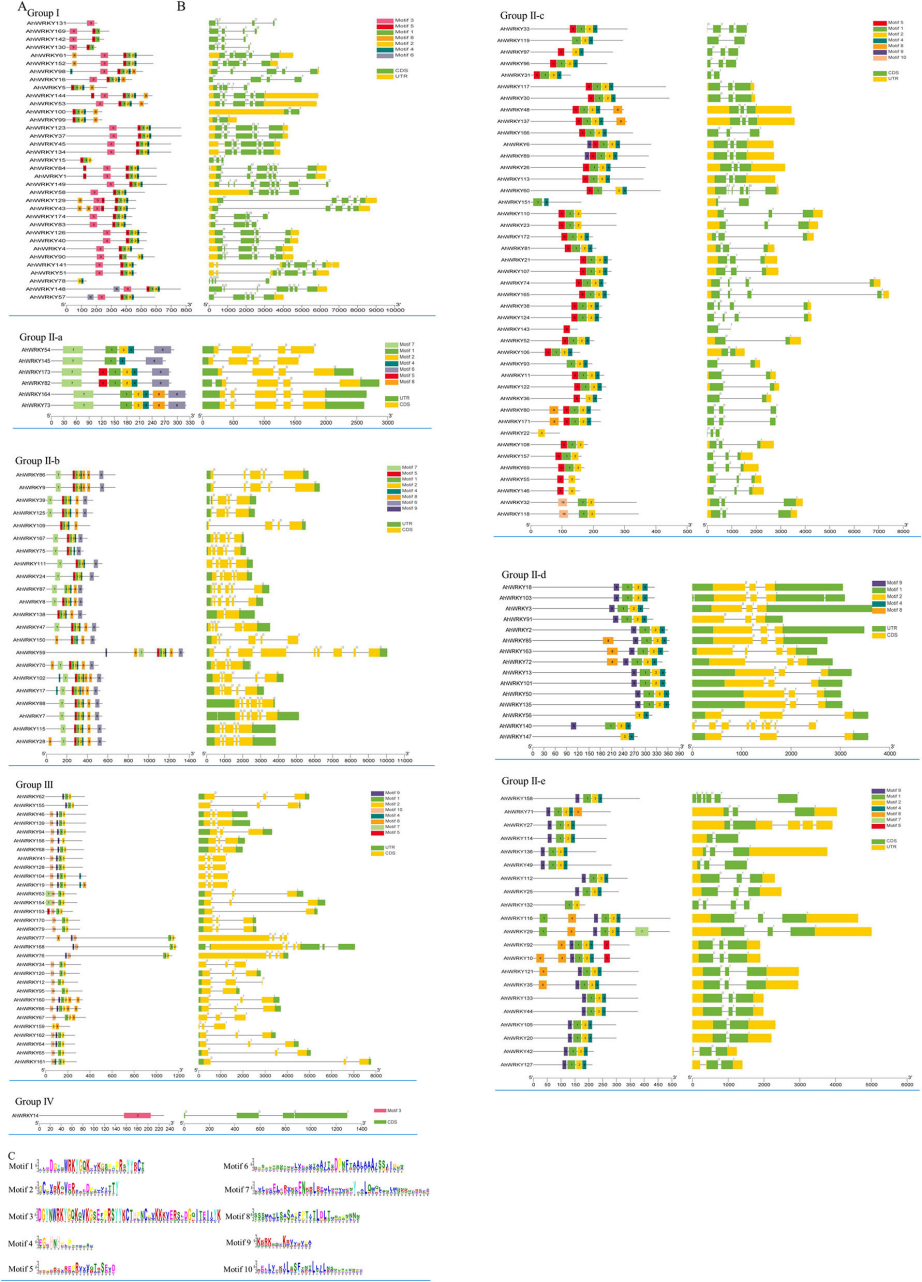
Gene structures and conserved motif analysis of *AhWRKY* genes. **(A)** The conserved motif of AhWRKY. **(B)** The exon–intron structure of AhWRKY. **(C)** The amino acid composition of each motif. Each conserved motif is represented by various-colored rectangles. Box length corresponds to motif length. Different colors represent different group members. Due to the large image size, we have presented the structures and motifs in various group-wise.

The numbers of introns varied among 174 *AhWRKY* genes. All the *AhWRKY* family genes contain one to eight introns. Most members of group I and subgroup II-b have four introns, whereas most members of subgroup II-c, subgroup II-d, subgroup II-e, and group III have two introns. Four members of subgroup II-a have three introns, and two members have four introns. The member of group IV has two introns (Figures 4A,B; Supplementary Table 2). There was some subgroup specificity, possibly attributed to the number of changes of introns during evolution. In contrast, the number and position of the introns were relatively conserved in the same group of plant species (Figures 4A,C).

Ten conserved motifs of 174 *AhWRKY* genes were analyzed using the MEME software (Figures 4A–C). Motif 1 was dominantly present in the WRKY domain regions of most family members. The proteins of the same group showed identical numbers and arrangements of motifs, five motifs were detected in group I (motifs 3, 5, 1, 2, and 4), motif 3 in the N terminal, and motif 1 in the C terminal contained WRKY. Five and seven motifs were detected in subgroup II-a (motifs 7, 1, 2, 4, and 6) and II-b (motifs 7, 5, 1, 2, 4, 8, and 6), four motifs in subgroup II-c (motifs 5, 2, 1, and 4), II-d (motifs 9, 2, 1, and 4) and II-e (motifs 9, 2, 1, and 4), three motifs in group III (motifs 9/10, 1, and 2), and one motif in group IV (motif 3). Motifs in the same group showed great similarity, indicating the functional conservation in different groups. Comparing the intron–exon structure and conserved motif analysis, members of the same group showed great similarity in characteristics, indicating that most *AhWRKY* genes were highly conserved among groups.

### Prediction analysis of *cis*-acting elements with *AhWRKY*

**Figure 5:**
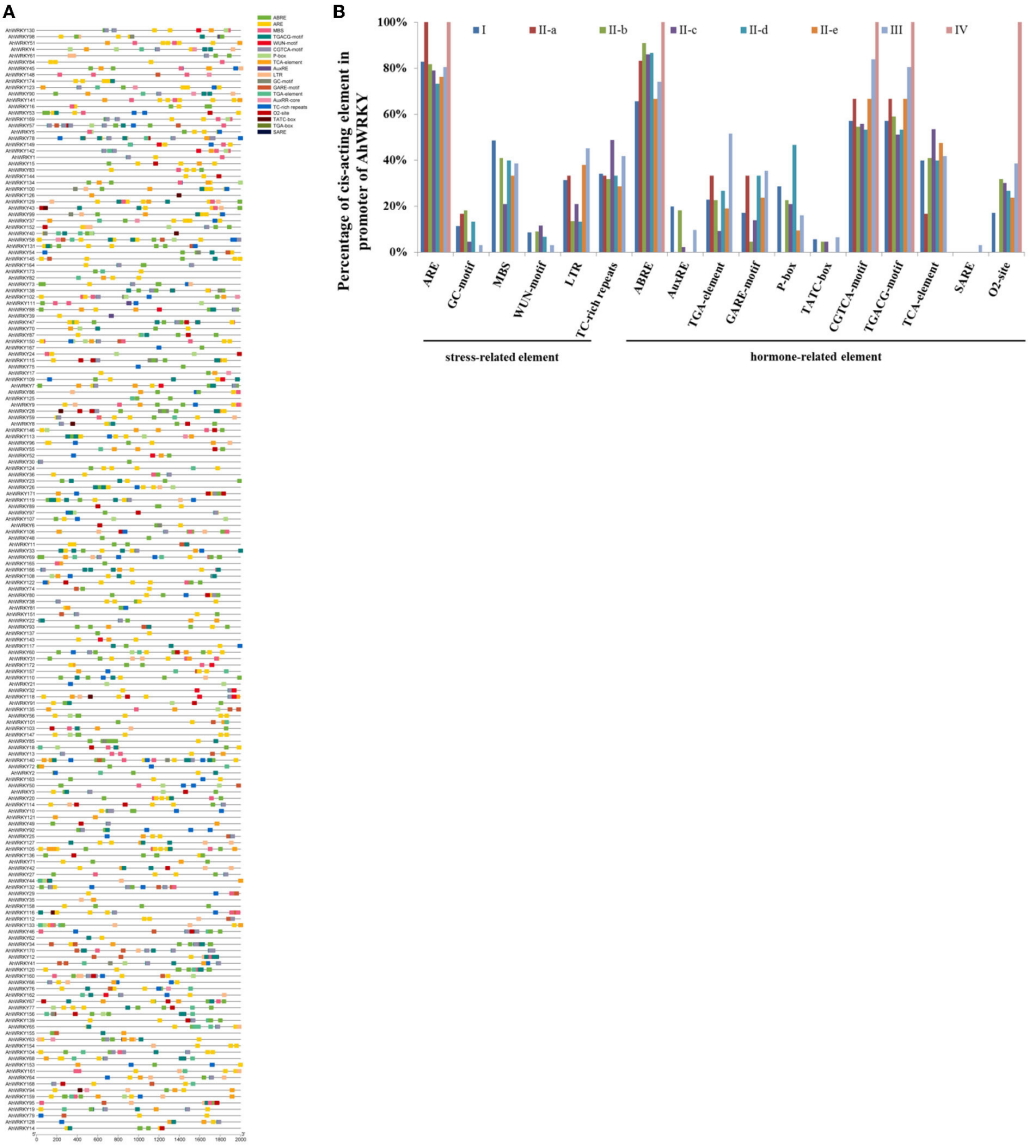
Identification of the cis-acting element in the promoter of *AhWRKY* genes. **(A)** Each type of element is represented by a number of colored rectangles. Box length corresponds to element length. Different colors represent different group members. **(B)** Percentage of each cis-acting element in the promoter of the AhWRKY.

To further study the potential regulatory mechanism of the *AhWRKY* genes, the 2 kb upstream sequence of the *AhWRKY* genes translation start site was used to detect the *cis*-elements. A total of 55 known *cis*-elements (31 light-related elements, 11 hormone-related elements, seven tissue-specific elements, and six stress-related elements) were detected (Figure 5). MBS (drought inducibility), TC-rich repeats (defense and stress responsiveness), WUN motif (wound responsiveness), LTR (low-temperature responsiveness), ARE (essential for the anaerobic induction), and GC motif (anoxic specific inducibility) involved in stress responses are found in 52.5, 2.9, 14.9, 2.6, 12.1, and 15% of AhWRKY promoters, respectively. Meanwhile, there are a large number of hormonal response elements, including ABRE, AuxRR, and TGA-element, CGTCA motifs and TGACG-motif, GARE motifs, P-box, and TATC-box, O2 site, TCA element, and SARE. Promoters of AhWRKY possess a relatively large number of ARE elements, 83, 100, 82, 78, 73, 76, 81, and 100% family members of the group I, II-a, II-b, II-c, II-d, II-e, III, and IV possessed ARE elements. Notably, 20% of family members of group I possessed AuxRE, 91% of family members of subgroup II-b possessed ABRE elements, 54 and 48% of family members of subgroup II-c and II-e possessed TCA-element, 47% of family members of subgroup II-d possessed P-box, 84% family members of group III possessed CGTCA-motif. There was a divergence in *cis*-acting elements in promoter regions of different groups.

### Expression of *AhWRKY* in different tissues of peanuts

**Figure 6:**
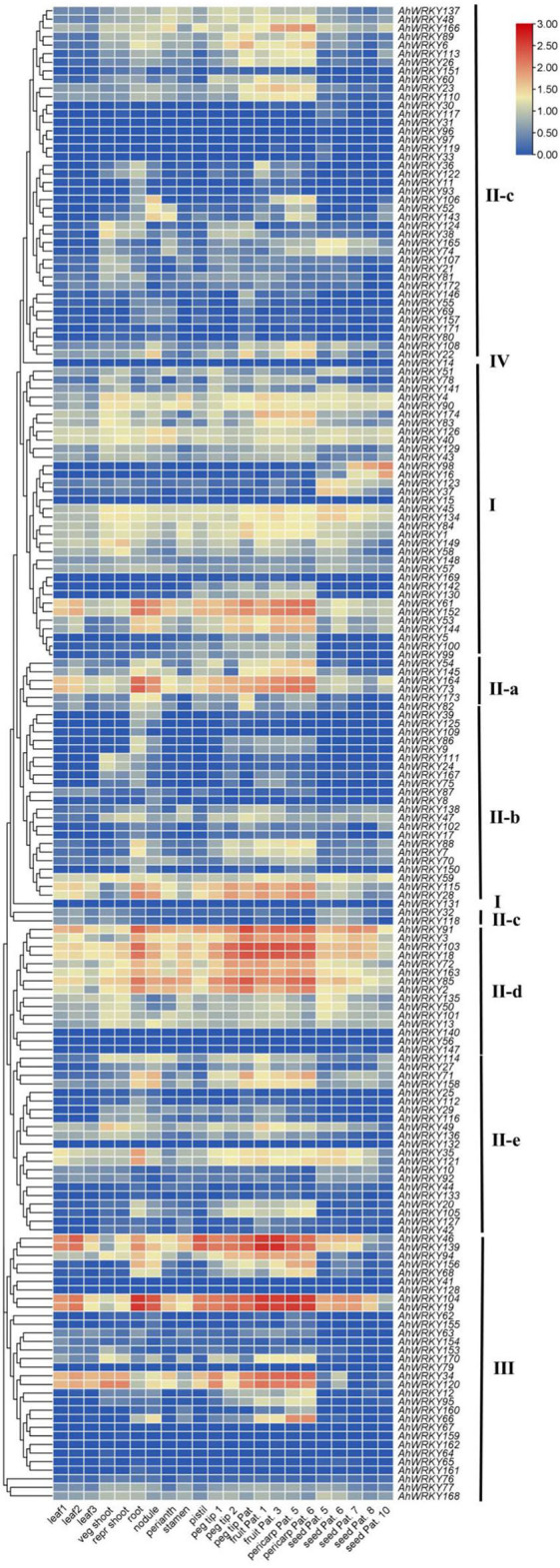
Expression profiles of *AhWRKY* genes across the different tissues. Gene expression is expressed in l g (TPM+1). Seedling leaf 10 days post-emergence (leaf 1), main stem leaf (leaf 2), lateral stem leaf (leaf 3), vegetative shoot tip from the main stem (veg shoot), reproductive shoot tip from first lateral (repr shoot), 10-day roots (root), 25-day nodules (nodule), aerial gynophore tip (peg tip 1), subterranean peg tip (peg tip 2), Pattee 1 stalk (peg tip Pat. 1), Pattee 1 pod (fruit Pat. 1), Pattee 3 pod (fruit Pat. 3), Pattee 5 pericarp (pericarp Pat. 5), Pattee 6 pericarp (pericarp Pat. 6), Pattee 5 seed (seed Pat. 5), Pattee 6 seed (seed Pat. 6), Pattee 7 seed (seed Pat. 7), Pattee 8 seed (fruit Pat. 8), and Pattee 10 seed (seed Pat. 10).

Generally, the gene expression had a similar pattern within the same phylogenetic classes. Members of group I showed high gene expression levels in peg tip Pat, fruit Pat. 1, fruit Pat. 3, pericarp Pat. 5, and pericarp Pat. 6, and two members *(AhWRKY61* and *AhWRKY152*) were also highly expressed in root and nodule. The expression pattern of members of subgroup II-a was similar to group I, and two members (*AhWRKY163* and *AhWRKY74*) showed the same trend as *AhWRKY61* and *AhWRKY152*. Genes of subgroup II-b showed low expression levels in all tissues, but *AhWRKY115* and *AhWRKY28* had the same expression trend as *AhWRKY61* and *AhWRKY152*. Members of subgroup II-c were highly expressed in fruit Pat. 3, pericarp Pat. 5, and pericarp Pat. 6, most members of subgroup II-d were highly expressed in all tissues, and most members of subgroup II-e and group III were less expressed in all tissues (Figure 6).

### *AhWRKYs* gene expression in response to *Ralstonia solanacearum* infection

**Figure 7:**
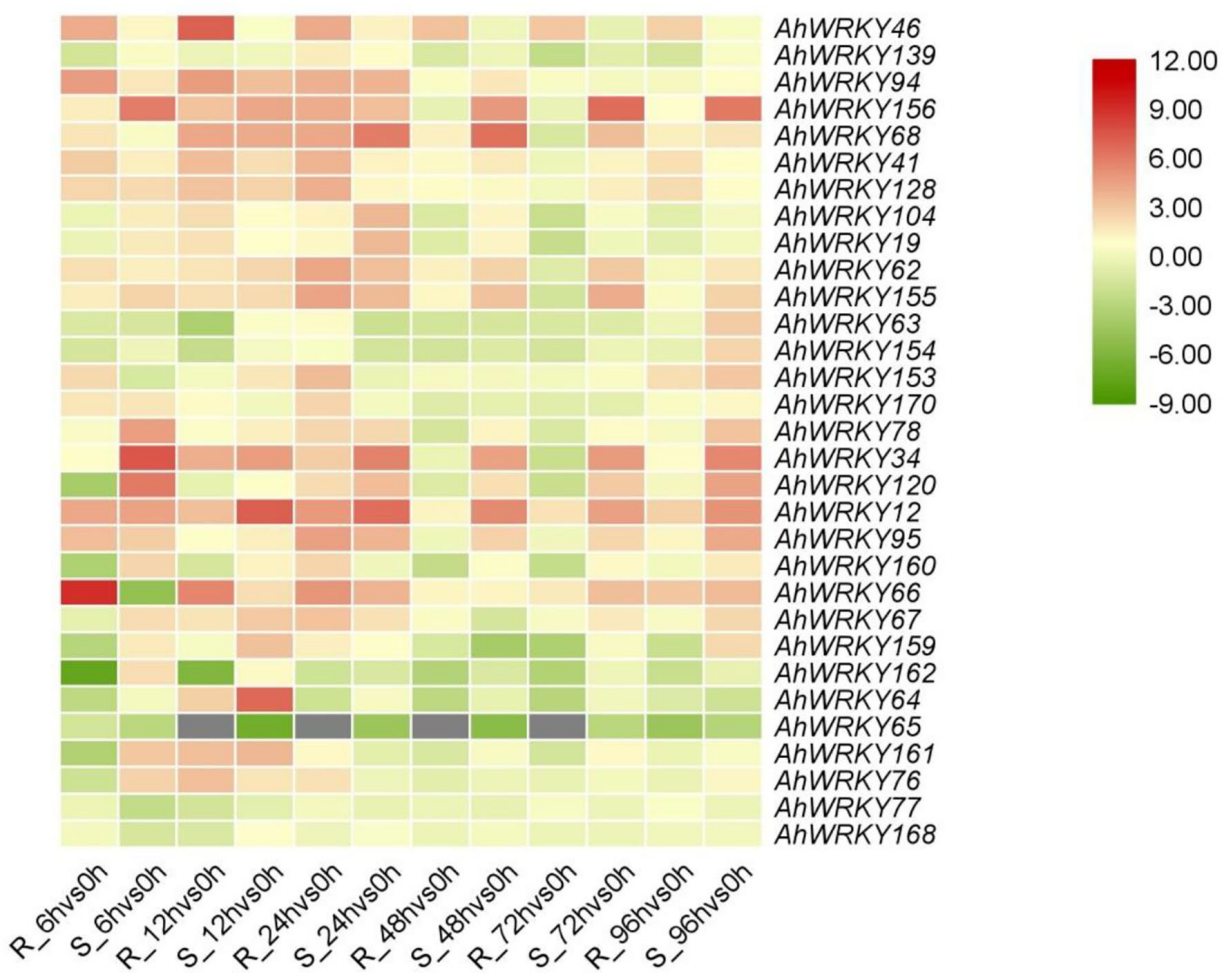
Expression of *AhWRKYs* in the two cultivars infected with *Ralstonia solanacearu*m. The qRT-PCR of each gene in peanut roots infected with *Ralstonia solanacearu*m in the two cultivars is shown. Heatmap showed the log2-transformed ratio values. R, Resistance; S, Susceptibility.

The expression patterns of *AhWRKY* genes in group III differed between the two cultivars in response to *R. solanacearum* infection (Figure 7). One and six members of group III showed the highest and lowest expression in 6 h, eight and five in 12 h, and 13 highest in 24 h after infection with *R. solanacearum* in ganhua18. Four and eighth members of group III showed the highest and lowest expression in 6 h, seven and one in 12 h, six and three in 24 h, one highest expressed in 48 and 72 h, and five highest expressed in 96 h after infection with *R. solanacearum* in kanong313. *AhWRKY139, AhWRKY63, AhWRKY154, AhWRKY120, AhWRKY160, AhWRKY159, AhWRKY162, AhWRKY64, AhWRKY65*, and *AhWRKY161* were downregulated in ganhua18 after *R. solanacearum* infection. Whereas, *AhWRKY63, AhWRKY154, AhWRKY153, AhWRKY66, AhWRKY67, AhWRKY159, AhWRKY162, AhWRKY65, AhWRKY77*, and *AhWRKY161* were downregulated in kainong313 after *R. solanacearum* infection, these genes may negatively regulate bacterial wilt resistance in peanuts. Moreover, *AhWRKY46, AhWRKY94, AhWRKY156, AhWRKY68, AhWRKY41, AhWRKY128, AhWRKY104, AhWRKY19, AhWRKY62, AhWRKY155, AhWRKY153, AhWRKY170, AhWRKY78, AhWRKY34, AhWRKY12, AhWRKY95, AhWRKY66, AhWRKY67*, and *AhWRKY76* were upregulated in ganhua18 after *R. solanacearum* infection. On the other hand, *AhWRKY46, AhWRKY94, AhWRKY156, AhWRKY68, AhWRKY41, AhWRKY128, AhWRKY104, AhWRKY19, AhWRKY62, AhWRKY155, AhWRKY170, AhWRKY78, AhWRKY34, AhWRKY120, AhWRKY12, AhWRKY95, AhWRKY160, AhWRKY64, AhWRKY161*, and *AhWRKY76* were upregulated in kainong313 after *R. solanacearum* infection, indicating that these genes may positively regulate bacterial wilt resistance in peanuts (Figure 7).

After 6 h of *R. solanacearum* infection, the expressions of *AhWRKY120, AhWRKY159, AhWRKY161*, and *AhWRKY76* were downregulated by 0.07, 0.13, 0.1, and 0.28 than control in ganhua18, while that in kainong313 were increased by 66.91, 3.12, 8.19 and 6.05 than control. After 72 h of infection with the *R. solanacearum*, the expressions of *AhWRKY155, AhWRKY78*, and *AhWRKY34* were downregulated by 0.34, 0.45, and 0.24 than control in ganhua18, while that in kainong313 were increased by 17.46, 2.04, and 27.58 than control. After 6 h and 12 h infection with the *R. solanacearum*, the expression of *AhWRKY162* was downregulated by 0.01 and 0.02 than control in ganhua18, while that in kainong313 was increased by 4.35 and 2.11 than control. After 6 h, 12 h, and 72 h infection with the *R. solanacearum*, the expression of *AhWRKY160* was downregulated by 0.09, 0.39, and 0.21 than control in ganhua18, while that in kainong313 was increased by 5.64, 2.56, and 2.17 than control. After 6 h infection with the *R. solanacearum*, the expression of *AhWRKY153* was upregulated by 5.03 than control in ganhua18, while that in kainong313 was reduced by 0.41 than control (Figure 7). The differences in bacterial wilt resistance of two cultivars may be attributed to the expression patterns of these genes.

### miRNA targeting *AhWRKY* genes

**Figure 8:**
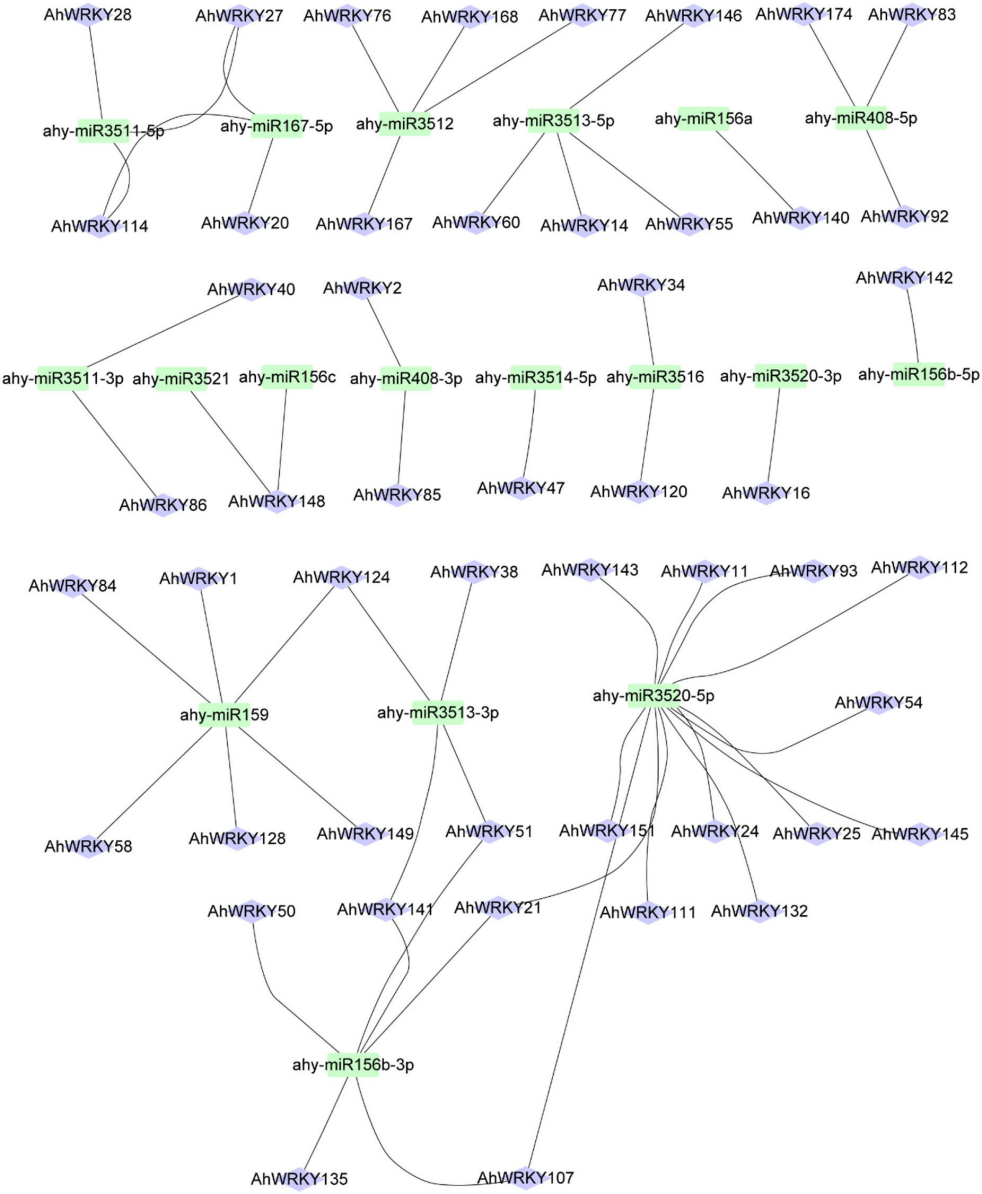
Network figure of identified miRNA targeting *AhWRKY* genes. Green box colors correspond to miRNAs, and bluish ellipse shapes represent target *WRKY* genes.

This study forecasted 18 miRNAs targeting 50 *AhWRKY* genes (Figure 8; Supplementary Table 3). The comprehensive data of all miRNAs targeted genes/sites is given in Supplementary Table 3. Overall, the results established that ahy-miR3520-5p target a maximum number of genes (13), followed by ahy-miR159 and ahy-miR156b-3p which targets six *AhWRKY* genes (Figure 8). Whereas, some genes are only targeted by one miRNA, such as *AhWRKY47, AhWRKY16, AhWRKY142*, and *AhWRKY140* (Figure 8). In further investigations, the transcript levels of these miRNAs and their targeted genes need confirmation to govern their biological acts in peanut breeding.

## Discussion

As one of the most important and largest TF, WRKY TFs play a critical role in response to biotic stresses in plants (Rushton et al., 2010; Javed et al., 2020, 2022). WRKY families have been reported in many plants, but little is known regarding the functions of the WRKY family in *A. hypogaea*. There were 77, 75, and 158 WRKY proteins in *Arachis duranensis, Arachis ipaënsis*, and *Arachis hypogaea* with reference to the first version of the *A. hypogaea* genome, respectively (Song et al., 2016; Zhao et al., 2020). Based on the first and second versions of the *A. hypogaea* genome released on June 4, 2021, this study identified 174 *AhWRKY* genes in peanuts, 83 *WRKY* genes in the A genome, and 91 in the B genome. In our study, there were 17 more genes in *A. hypogea* genome and one fewer genes in A and B genomes than previous reports. Previous studies analyzed the expressions of *AhWRKY* under drought stress in peanuts (Zhao et al., 2020); however, this study analyzed expressions of *AhWRKY* genes in response to *R. solanacearum* infection in peanuts. The high multiplicity/unpredictability in the quantity of WRKY TFs among diverse plant species could be due to unalike evolutionary events or duplication of whole genomes during the evolutionary period. In particular, the distinctive reports of replicated (tandem/segmental) genes succeeding in the repetition of entire genomes could also be accountable for continuing evolutionary changeovers and crop domestication (Leister, 2004; Bertioli et al., 2016, 2019; Van De Peer et al., 2017; Bohra et al., 2022). Nevertheless, previous reports also validated that segmental and tandem repetitions, particularly for the previous events, could be important driving factors in the evolution and enlargement of WRKY TFs in diverse crop plants (Song et al., 2016; Xie et al., 2018; Zhao et al., 2020; Javed et al., 2022).

Mutations and deletions prevail in the plant WRKY domain (Wei et al., 2012). Five *WRKY* genes deleted one WRKY domain in *Helianthus annuus* L. (Li J et al., 2020). Nine *WRKY* genes deleted one WRKY domain, seven *WRKY* genes deleted zinc finger, and three *WRKY* genes deleted one WRKY motif in maize (Hu et al., 2021). Previous reports suggest that mutations also occurred in the WRKY motif of most *WRKY* genes in peanuts (Song et al., 2016; Zhao et al., 2020). In this study, there were 17 *AhWRKYs* mutated and 13 *AhWRKYs* deleted in the WRKY domain. One group I member *AhWRKY* motif changed to WRKYGEK. Seven members of subgroup II-c *AhWRKY* motif changed to WRKYGKK, and two members changed to WRKYGEK, two members changed to WRKYGRK. WRKY motif of *AhWRKY36* (subgroup II-c), *AhWRKY131* (I), *AhWRKY97* (subgroup II-e), and *AhWRKY14* (IV) changed to WRK, WRMYGQK, WHKYGKK, and GRKYGQK, respectively. Moreover, 12 *AhWRKYs* mutated and deleted in zinc fingers. Zinc finger deleted in C terminal of *AhWRKY130* (group I) and N terminal of *AhWRKY169* (group I). Zinc finger deleted in *AhWRKY145* (subgroup II-a), *AhWRKY75* (subgroup II-b), *AhWRKY150* (subgroup II-b), *AhWRKY9* (subgroup II-c), *AhWRKY143* (subgroup II-c), and *AhWRKY151* (subgroup II-c). Zinc finger changed to C-X4-C-X25-H-X-S in *AhWRKY22* (subgroup II-c), C-X5-C in *AhWRKY132* (subgroup II-e), C-X4-C-X22-H-X-Y in *AhWRKY14* (IV), and C-X4-C in *AhWRKY131* (I). There are many mutations in the zinc finger of group I and subgroup II-e and the WRKY motif of subgroup II-c presumed that the variation in members of group I, subgroup II-c, and II-e generate new functions.

Gene duplication plays an important role in the amplification and evolution of plant gene families (Cannon et al., 2004; Leister, 2004; Bertioli et al., 2016; Van De Peer et al., 2017). Among gene clusters, five pairs of tandem duplication and nine pairs of segmental duplication genes were identified; seven pairs of tandem duplication and 132 pairs of segmental duplication genes were identified in 14 and 144 *AhWRKYs* (Figure 3), which were one more tandem duplication and nine segmental duplications than the previous study (Zhao et al., 2020). The divergence of two wild ancestral diploids of cultivated tetraploid peanuts may occur in 2.16 MYA (Chen et al., 2019b). Ninety-nine pairs of segmental duplication genes occurred before the divergence of two wild ancestral diploids, 31 pairs of tandem duplication, and six pairs of segmental duplication genes occurred after the divergence of two wild ancestral diploids. The ka/ks ratios of 99.4% (173/174) of *AhWRKYs* were less than one, which suggested that most *AhWRKYs* were selected for purification. The ka/ks ratios of one gene pair, including *AhWRKY119* and *AhWRKY33*, was 1.21 (more than 1), which indicated that these genes were in a state of positive selection in peanuts, evolving rapidly, and might be very important for the evolution of peanuts.

Prediction analysis of *cis*-acting elements with *AhWRKYs* indicated many light-related, hormone-related, tissue-specific, and stress-related elements in the promoters of *AhWRKY* genes (Figure 5), which was in accordance with the previous study (Zhao et al., 2020) and speculated that *AhWRKY* is involved in various biological processes.

Tissue-specific expression suggested that genes from the same group indicated similar expression patterns (Figure 6). Tissue-specific expression was in agreement with the tissue-specific elements in the promoters of *AhWRKY* genes. For instance, *AhWRKY16* was highly expressed in seed, and an RY-element, which is a *cis*-acting regulatory element involved in seed-specific regulation in the promoter, and *AhWRKY158* was highly expressed in nodule and a nodule-site1 element, which is a nodule-specific factor binding site in its promoter (Figures 5, 6).

LRR is closely related to the disease resistance of plants (Jones and Dangl, 2006; McHale et al., 2006). It is known that some WRKY proteins contain the NBS-LRR domain (Deslandes et al., 2003; Shen et al., 2007; Liu et al., 2016), and WRKY proteins from group III are mainly involved in plant disease resistance (Kalde et al., 2003; Eulgem and Somssich, 2007). *AtWRKY52* possessed the NBS-LRR domain and WRKY domain, which is a critical gene in regulating bacterial wilt resistance (Deslandes et al., 1998, 2002). *CaWRKY58* and CaWRKY40b (II-a) negatively regulate bacterial wilt resistance, while *CaWRKY22* (II-e) and *CaWRKY27* (II-e), *CaWRKY28* (II-c), *CaWRKY30* (III), and *CaWRKY41* (III) positively regulate bacterial wilt resistance in pepper (Wang et al., 2012; Dang et al., 2014, 2019; Hussain et al., 2018, 2021; Muhammad et al., 2018; Yang et al., 2021). *SiWRKY53* (III) responded to the bacterial wilt resistance (Mishra et al., 2021). WRKY members of group III played a vital role in plant bacterial wilt resistance. In this study, *AhWRKY76* and *AhWRKY77* of group III had LRR domain. qRT-PCR results showed that *AhWRKY77* was downregulated by 0.34 than control in 12 h of ganhua18 and 0.19 than control 6 h of kainong313 after infection with the *R. solanacearum*, which may negatively regulate bacterial wilt resistance in peanuts. After *R. solanacearum* infection, *AhWRKY76* displayed lower-higher expressions, i.e., 0.28, 10.35, and 3.97 than control in 6 h, 12 h, and 24 h of ganhua18, respectively, and higher expressions by 6.05 and 3.53 than control in 6 h and 12 h in kainong313, respectively, which may contribute to explain the difference in BW resistance between the two cultivars. These outcomes suggest that *AhWRKY76* and *AhWRKY77* may play a critical role in bacterial wilt resistance in peanuts. *AhWRKY76* and *AhWRKY77* targeted by ahy-miR3512 showed that ahy-miR3512 might have a vital role in BW resistance (Figure 7).

## Conclusion

In conclusion, a total of 174 *AhWRKY* genes were identified in *A. hypogaea*. The classification, chromosomal location, collinearity, protein structure, conserved motif composition, *cis*-acting elements, putative miRNAs, and phylogenetic relationship of *AhWRKYs* were systematically analyzed, which provide a basis for further study of the molecular and functional structure of *AhWRKYs*. Furthermore, the expression patterns of tissues-specific and pathogen-responsive *AhWRKYs* were obtained from RNA-seq data and qRT-PCR results, providing useful information for further functional investigations in response to biotic stresses. Ten AhWRKY genes (*AhWRKY34, AhWRKY76, AhWRKY78, AhWRKY120, AhWRKY153, AhWRKY155, AhWRKY159, AhWRKY160, AhWRKY161*, and *AhWRKY162*) from group III displayed different expression patterns in sensitive and resistant peanut genotypes infected with the *R. solanacearum*. Two AhWRKY proteins (AhWRKY76 and AhWRKY77) from group III obtained the LRR domain; *AhWRKY77* was downregulated in two cultivars, and *AhWRKY76* showed lower-higher expression ganhua18 and higher expression in kainong313. Both *AhWRKY76* and *AhWRKY77* were targeted by ahy-miR3512, which may have an important function in peanut disease resistance. The positively upregulated genes could be used for further characterization in peanuts to develop disease-smart peanut cultivars. In short, our study could help researchers better understand the function and regulatory mechanism of *AhWRKYs* genes in *A. hypogaea* during pathogen response.

## Data availability statement

The datasets presented in this study can be found in online repositories. The names of the repository/repositories and accession number(s) can be found in the article/Supplementary material.

## Author contributions

LY carried out the bioinformatic analysis and drafted the manuscript. LY and HJ collected the plant materials and performed the experiments. AR helped with some analysis and improved the manuscript. LY, AR, and YH participated in handling figures and tables. LY, AR, DG, and XZ edited and revised the manuscript. All authors read and approved the final version of the manuscript.

## Funding

This work was supported by the Agricultural Collaborative Innovation Project of Jiangxi Province, China (JXXTCXBSJJ2022007 and JXXTCX202112), and the Third Census and Collection of Crop Germplasm Resources in China.

## Conflict of interest

The authors declare that the research was conducted in the absence of any commercial or financial relationships that could be construed as a potential conflict of interest.

## Publisher's note

All claims expressed in this article are solely those of the authors and do not necessarily represent those of their affiliated organizations, or those of the publisher, the editors and the reviewers. Any product that may be evaluated in this article, or claim that may be made by its manufacturer, is not guaranteed or endorsed by the publisher.
